# Strengthening organizational performance through accreditation research-a framework for twelve interrelated studies: the ACCREDIT project study protocol

**DOI:** 10.1186/1756-0500-4-390

**Published:** 2011-10-09

**Authors:** Jeffrey Braithwaite, Johanna Westbrook, Brian Johnston, Stephen Clark, Mark Brandon, Margaret Banks, Clifford Hughes, David Greenfield, Marjorie Pawsey, Angus Corbett, Andrew Georgiou, Joanne Callen, John Øvretveit, Catherine Pope, Rosa Suñol, Charles Shaw, Deborah Debono, Mary Westbrook, Reece Hinchcliff, Max Moldovan

**Affiliations:** 1 University of New South Wales, Kensington, NSW 2052, Australia; 2 Australian Council on Healthcare Standards, 5 Macarthur Street, Ultimo NSW 2007, Australia; 3 Quality in Practice/Australian General Practice Accreditation Limited, PO Box 2058, Milton QLD 4064, Australia; 4 Aged Care Accreditation Agency Limited, PO Box 773, Parramatta NSW 2124, Australia; 5 Australian Commission on Safety and Quality in Health Care, GPO Box 5480, Sydney NSW 2001, Australia; 6 Clinical Excellence Commission, GPO Box 1614, Sydney, NSW 2001, Australia; 7 University of Technology, 15 Broadway, Ultimo NSW 2007, Australia; 8 Karolinska Institute, Fakturor, Box 23 109, SE-104 35 Stockholm, Sweden; 9 University of Southampton, University Road, Southampton SO17 1BJ UK; 10 Avedis Donabedian University Institute, Autonomous University of Barcelona, CIBER Epidemiology and Public Health (CIBERESP), Spain; 11 European Society for Quality in Healthcare, St Camillus Hospital, Shelbourne Road, Limerick, Ireland

## Abstract

**Background:**

Service accreditation is a structured process of recognising and promoting performance and adherence to standards. Typically, accreditation agencies either receive standards from an authorized body or develop new and upgrade existing standards through research and expert views. They then apply standards, criteria and performance indicators, testing their effects, and monitoring compliance with them. The accreditation process has been widely adopted. The international investments in accreditation are considerable. However, reliable evidence of its efficiency or effectiveness in achieving organizational improvements is sparse and the value of accreditation in cost-benefit terms has yet to be demonstrated. Although some evidence suggests that accreditation promotes the improvement and standardization of care, there have been calls to strengthen its research base.

In response, the ACCREDIT (**A**ccreditation **C**ollaborative for the **C**onduct of **R**esearch, **E**valuation and **D**esignated **I**nvestigations through **T**eamwork) project has been established to evaluate the effectiveness of Australian accreditation in achieving its goals. ACCREDIT is a partnership of key researchers, policymakers and agencies.

**Findings:**

We present the framework for our studies in accreditation. Four specific aims of the ACCREDIT project, which will direct our findings, are to: (i) evaluate current accreditation processes; (ii) analyse the costs and benefits of accreditation; (iii) improve future accreditation via evidence; and (iv) develop and apply new standards of consumer involvement in accreditation. These will be addressed through 12 interrelated studies designed to examine specific issues identified as a high priority. Novel techniques, a mix of qualitative and quantitative methods, and randomized designs relevant for health-care research have been developed. These methods allow us to circumvent the fragmented and incommensurate findings that can be generated in small-scale, project-based studies. The overall approach for our research is a multi-level, multi-study design.

**Discussion:**

The ACCREDIT project will examine the utility, reliability, relevance and cost effectiveness of differing forms of accreditation, focused on general practice, aged care and acute care settings in Australia. Empirically, there are potential research gains to be made by understanding accreditation and extending existing knowledge; theoretically, this design will facilitate a systems view of accreditation of benefit to the partnership, international research communities, and future accreditation designers.

*"Accreditation of health-care organisations is a multimillion dollar industry which shapes care in many countries. Recent reviews of research show little evidence that accreditation increases safety or improves quality. It's time we knew about the cost and value of accreditation and about its future direction." *[Professor John Øvretveit, Karolinska Institute, Sweden, 7 October 2009]

## Background

**Figure 1:**
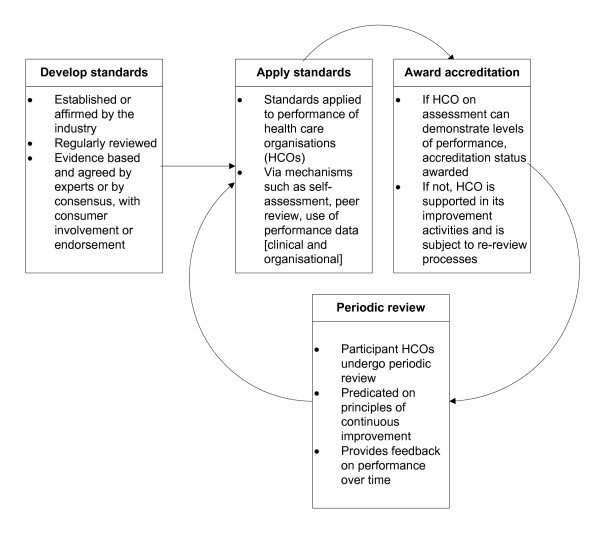
**Generic accreditation model**.

Service accreditation is a system of organizational improvement centred on a certifying agency (or accrediting body) assessing performance against pre-determined standards, usually by multiple means. Internationally, accreditation is designed to improve organizations by developing new standards or upgrading existing standards through research or expert advice, and by defining criteria and performance indicators and applying these standards, criteria and indicators to organizational processes and outcomes. Although models differ in detail, [1] most accreditation systems assess and rate the performance of organizations and services by evaluating their progress and appraising their compliance with standards, usually via mechanisms such as self-assessment surveys, data review and structured visits by surveyors. Some systems use peer surveyors and others persons whose background is audit methodology. Following training, assessors or surveyors have detailed knowledge of applicable standards. Figure 1 provides a generic accreditation model which illustrates a typical accreditation process from standards development into the cycle of standards application, assessment and award of accreditation and periodic review.

The reach of accreditation is extensive and the investments in it in many sectors are considerable. Industries such as school education, [2] universities, [3,4] software manufacture, [5] the seafood sector, [6] and ambulance services [7] have embraced accreditation, standard setting, and surveying processes. Accreditation has been applied to laboratories, [8] management systems, [9] products,[10] medical curricula, [11] and staff competencies [12]. Essentially, the core concerns addressed by the processes of accreditation are whether organizations satisfy pre-designated standards, are regularly examined and continuously improved, and the extent to which customer satisfaction is met or enhanced [13]. However, we lack convincing evidence of the long-term effects and organizational impact of accreditation processes.

The health sector, as an early adopter of accreditation, has promoted its use since 1951 (the Joint Commission in the United States of America), and in Australia since 1973. Stakeholders recognize its potential to improve organizational performance, quality of care, safety standards and consumer satisfaction. However, despite support for accreditation among informed groups, accreditation has had its share of criticism, including the lack of supporting evidence and concerns about the costs of uncertain benefits and whether it offers value for money [14-16]. There is a desire among stakeholders to strengthen the research base. This project is a response to the need for a program of research into accreditation that links the key industry partners and policy bodies with interested researchers, and plans to produce results which will link with other multi-method, multi-phased studies underway in Europe [17].

The ACCREDIT (**A**ccreditation **C**ollaborative for the **C**onduct of **R**esearch, **E**valuation and **D**esignated **I**nvestigations through **T**eamwork) project is a partnership led by researchers in the Centre for Clinical Governance Research and Centre for Health Systems and Safety Research in the Australian Institute of Health Innovation (AIHI) at University of New South Wales with the three major Australian health-sector accreditation agencies (The Australian Council on Healthcare Standards [ACHS], Australian General Practice Accreditation Limited [AGPAL], and Aged Care and Standards Accreditation Agency [ACSAA]), the leading quality improvement policy bodies (the Australian Commission on Safety and Quality in Health Care [ACSQHC] and the Clinical Excellence Commission [CEC]), key Australian investigators, and international collaborators. These partners are dedicated to studying the impact of accreditation and to executing an extended research program, to provide evidence and empirical models for ways in which accreditation can be improved.

### Research significance and importance of the problem

Some evidence suggests that accreditation programs can promote change [18] and the standardization of services and organizational processes, including how decisions about care are made [19]. However, the research literature is either inconsistent or does not support the contentions that accreditation directly improves organizational performance, quality of care, and patient satisfaction [14,20-22]. In one of the first studies to attempt to link accreditation with organizational outcomes, we found that accreditation was significantly positively correlated with organizational culture (*P *= 0.005) and leadership (*P *= 0.005), but there was weaker statistical evidence on the relation to clinical indicator performance (*P *= 0.080) [23]. No statistically significant association was observed between accreditation and organizational climate (*P *= 0.110) or consumer involvement (*P *= 0.377) [23].

Thirty-four of 89 selected hospitals in the European Methods of Assessing Response to Quality Improvement Strategies (MARQuIS) project [24] were accredited (without International Organization for Standardization (ISO) certification), 10 were ISO9000-certificated without accreditation and 27 had neither accreditation nor certification. On 229 criteria of quality and safety, percentage scores were 66.9, 60.0 and 51.2 respectively. These statistically significant differences suggest that accreditation is a key quality strategy. However, there were confounding factors and a small sample, and the study did not substantially differentiate between accreditation and certification only [25].

To date, work on the costs and benefits of accreditation has been rudimentary [26-28]. Unless the economic benefits are modelled, we cannot make sound policy decisions about the future enhancement of accreditation, develop a new framework for its conduct, or understand its value.

In preparatory work to develop the framework reported here, we examined the literature concerning two initiatives that have recently received policy support: unannounced (short-notice) surveys conducted by surveyors [29] and tracer methodology (i.e., patient journeys) used to assess care [30]. We found no evidence for the benefits of short-notice surveys, whereas the limited studies of patient journeys suggested that they can be useful in evaluating care. Work commissioned by ACSQHC, and undertaken by ACHS, ACSAA and AGPAL in conjunction with ACCREDIT researchers assessing these short-notice surveys and patient journeys trials tentatively indicated that these can be useful tools which complement but do not substitute for existing methods.

**Figure 2:**
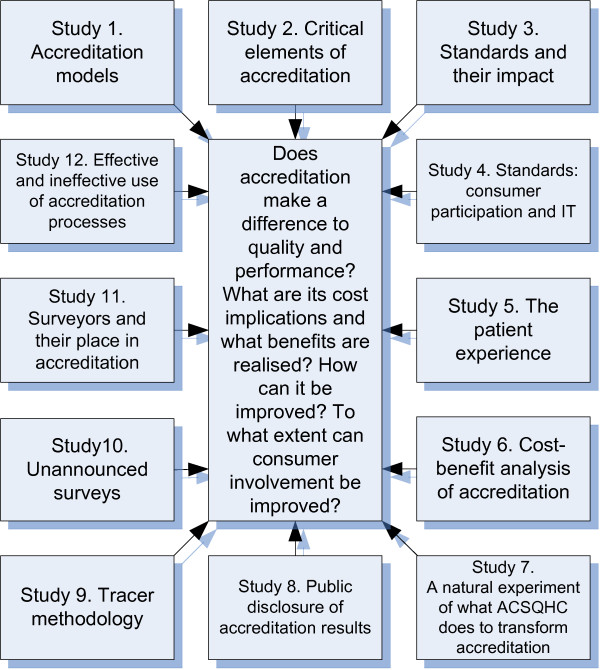
**Research strategy and studies**.

A systematic review of the literature conducted by the research team "... reveals a complex picture ... inconsistent findings ... [and] ... insufficient studies by which to draw conclusions."[[14] p.181] An overarching research framework with twelve interrelated studies (Figure 2) aims to address some of these gaps.

## Methods and design

### General aims

We are funded to execute a multi-method, triangulated research agenda with 12 studies designed by the ACCREDIT stakeholders. The ACCREDIT partners met in August 2007 to draft the conceptual framework and research plan. They subsequently refined the plan and conducted various studies, [14,20,23,26,31-33] evaluation projects, [29,30] literature reviews, [21,34,35] and partnership activities [36] to provide the empirical platforms for this proposal. An International Advisory Group offered strategic counsel to the project, and has an ongoing advisory role.

### Detailed research aims

The specific aims of the research address four main areas. These link 12 interrelated studies of issues identified as requiring research evidence as a high priority [14] (Table 1).

**Table 1:** Research aims, key questions, and related studies

Research aims	Key questions	Studies (Fig. 1)
1: To evaluate current accreditation processes	Does accreditation make a difference to the quality of care and performance?	1, 2, 3, and 12

2: To analyse the costs and benefits of accreditation	What are accreditation's cost implications? What benefits are realized?	6

3: To improve future accreditation	How can accreditation be improved?	7-11

4: To develop and apply new standards of consumer involvement in accreditation	How can consumer involvement be improved?	4 and 5

### Advancing the knowledge base

The research aims require a multi-method, [37] multi-level approach, [38] incorporating multi-layered data, [39] to provide rigorous answers to the key questions mapped to the four research aims (Table 1) and addressed in the 12 studies (Figure 1). The 12 studies are designed to answer questions to advance the accreditation knowledge base and meet expressed industry needs for empirical information. The proposal's research questions have emerged from extensive reviews [14,21,34] and consultations. Table 2 outlines the 12 interrelated studies that will address key derived questions, linking the 12 studies into four research aims.

**Table 2:** Twelve interrelated studies of accreditation--overview of approaches and methods

Study	Research questions	Research approaches, tasks, and scope	Methods, sample sizes, data requirements, analysis, design features
1. Accreditation models	What are the relative strengths and consequences of different accreditation models?	Undertake a multi-method evaluation of three accreditation models: those of the ACHS, AGPAL, and ACSAA	▪ Interview key stakeholders in three accreditation agencies (n = 18) ▪ Conduct a web-based questionnaire survey of acute health services, general practices and aged-care facilities (~n = 300)

2. Critical elements of accreditation	What are the critical elements of the accreditation process that stimulate improvement? What drives behaviour change in provider organizations and clinicians?	Assess each element (e.g., self-assessment, clinical indicators, patient data, surveyor visits and accreditation reports) and describe its role in promoting improvement	▪ Run focus groups of stakeholders drawn from accreditation agencies (n = 6 focus groups) and jurisdictional health departments (n = 8 focus groups), and 15 randomized focus groups from accredited general practices (n = 5), acute-care health-care organizations (n = 5), and aged-care providers (n = 5) ▪ Conduct a ranking exercise by surveying large samples of staff from accredited organizations across acute, general practice, and aged-care settings (~n = 600) to ascertain the relative importance of the accreditation elements ▪ From this sample, gather perspectives on and examples of how the respective accreditation elements drive change

3. Standards and their impact	How are standards developed and used? How do standards incorporate evidence, and influence the expertise of clinicians, managers, and policy makers? How does the application of standards promote change in organizational performance and clinical practice?	Examine the development of standards and their application using widespread observational activities and surveys across different accreditation programmes, selecting and investigating a sample of standards during their development phase to determine the sources of the standards (e.g., public inquiries, adverse events, international guidelines), how they should be developed, and how they should be applied	▪ Undertake ethnographic observations of the development of standards, assessing their use of evidence and the engagement of stakeholder groups ▪ Conduct a survey of accredited organizations, investigating how standards are applied and how they promote change (~n = 600 respondents). From these data, identify for detailed analysis case study sites in which standards have promoted measurable change ▪ Conduct case studies (n = 5) of specified key standards (evaluation of care, documented policies, the quality improvement system, health records, infection control). Use these case studies to identify factors related to organizational change. Obtain organizational data to quantify the extent of this change

4. Key new standard for consumer participation	Can we develop and trial a standard for consumer participation?	Use the Delphi method to create and field test a standard for consumer participation in acute settings, general practice, and aged care	▪ Systematically review instances of consumer participation cited in the accreditation literature ▪ Assess the review's evidence as the basis for the standard ▪ Consult with stakeholders, using the Delphi approach, to secure agreement on the standard ▪ Apply the standard in the field (n = 30) ▪ Evaluate its use and efficacy with survey and qualitative methods

5. The patient experience	How do patient experiences vary across a range of settings with differing accreditation results?	Compare the ethnographic mapping results for a range of patient experiences in different accreditation settings against positive and negative accreditation results	▪ Extend the research into patient journeys to a larger trial that includes all three accreditation domains ▪ Review 60 randomly selected complex patient journeys in depth ▪ Compare these against the accreditation results from each of the participating organizations in which patient journeys were taken.

6. Cost-benefit analysis of accreditation	What are the benefits and costs of accreditation and the different accreditation models?	Design and apply a model for a health-economics cost-benefit analysis (CBA) of accreditation, including an examination of the factors that affect (e.g., drive or inhibit) costs and benefits. Assess the benefits and costs over time, modelled on Brent [42]	▪ Conduct a detailed analysis of the cost effectiveness of accreditation in the three domains, examining the extent to which the benefits outweigh the costs ▪ Express the benefits and costs in nett present value terms, and adjust for the time value of money ▪ Seek cost estimates from the partner agencies, and benefit estimates from a randomized sample (n = 30) of their member organizations ▪ Execute CBA modelling on this basis

7. A natural experiment examining what the ACSQHC does to transform accreditation	What changes have ensued from the initiatives of the ACSQHC?	Conduct a formative evaluation of the impact of ACSQHC's transformation of accreditation, particularly the impact of the comprehensive set of National Safety and Quality Healthcare Standards applied to high-risk services	▪ Evaluate this progress using formative evaluation techniques, in a partnership arrangement with ACSQHC ▪ Review the gains made in establishing a national co-ordinating body to implement accreditation reforms, including standards development, piloting, implementation, expanding accreditation into high-risk services, and co-ordination with regulating bodies

8. Public disclosure of accreditation results	Is it possible to develop and test an effective model for the public disclosure of accreditation results?	Examine extant methods of public disclosure of information in international contexts, and their relative impacts	▪ Identify in a literature review the different models of public disclosure (e.g., types, formats, and approaches) and compare web-based reports, newsletters to health-care organizations, local newspaper reports, and community meetings ▪ Undertake focus groups with members of the public to investigate views and strategies (n = 10 focus groups) ▪ Conduct trials, with each accreditation domain, of the three most-relevant disclosure models (n = 30 enrollees)

9. Patient journey methodology	What is the effect of the application of the patient journey methodology?	Map the use of the patient journey methodology under various circumstances	▪ Evaluate the utilization of the patient journey technique using ethnographic observations of four accreditation survey teams in each of the three accreditation domains (n = 12 survey teams) ▪ Apply this knowledge to these domains, extending earlier tests of the patient journey method [30] ▪ Triangulate the results and compare and contrast the patient journey method against survey outcomes using standards

10. Short-notice surveys	What is the effect of the application of short-notice surveys?	Examine the use of short-notice surveys under various circumstances, including variables such as points in the accreditation cycle and service type	▪ Evaluate ACSAA's experience of short-notice surveys using key informant interviews with ACSAA staff (n = 10) and randomly selected aged-care facilities (n = 15) ▪ Apply this knowledge to trials with ACHS and AGPAL, extending earlier tests of short-notice surveys [29] ▪ Evaluate the ACHS and AGPAL trials and triangulate the data with previous results

11. Surveyors and their place in accreditation	What are the roles, effectiveness, and reliability of surveyors?	Conduct an ethnographic analysis of surveyors and surveying processes, with a comparative analysis of the roles, effectiveness and reliability of surveyors in the three accreditation domains	▪ Analyse existing accreditation databases to assess the relationships between the judgements and survey outcomes of accreditation teams, to quantify the variation between the teams and surveyors ▪ Undertake experiments, using test scenarios, with the methods developed in earlier research to assess the surveyors' reactions to and consistency in differing accreditation situations [31]

12. Differentiate effective and ineffective uses of accreditation processes and methods to promote change	How do effective and less-effective organizations use accreditation levers to improve performance?	Undertake a comparative, randomized, stratified examination of effective and less-effective organizations and the ways they use accreditation to promote performance improvement, drawing upon the results of study 3.	▪ Examine randomly selected organizations, 20 in each accreditation domain (n = 60 organizations) ▪ Separate these organizations into a split sample of effective and less-effective organizations, judged by stakeholders' attributions and external organizational performance criteria ▪ Apply detailed case study methods to derive both quantitative and qualitative indicators; assess how the two samples use accreditation to improve performance and promote change

### Methods, sample sizes and design features

The samples for the quantitative studies will be based on sample size calculations that ensure sufficient power to answer the questions under investigation. Qualitative studies will involve sample sizes based on saturation methods.

As shown in Table 2 a wide range of research techniques have been designed and will be applied, including objective empirical measurements, ethnographic observations, focus groups, interviews, trials, ranking exercises, and questionnaire surveys, providing a rich database. This will help create the triangulation effect often missing in discrete, project-based research, which has often produced unrelated, fragmented, and incommensurate findings in the past. A systems approach both to the triangulated multi-method design and to interpreting the findings will be taken, facilitating an understanding of the complex knowledge base that twelve interrelated studies will bring.

## Discussion

We have established a partnership with the main health-care, general-practice, and aged-care accreditation providers in the country, thereby incorporating the major accreditation domains in the one overarching study. This has allowed us to design policy- and industry-relevant research, e.g., to evaluate current accreditation processes (aim 1: studies 1, 2, 3 and 12) and to improve future accreditation approaches (aim 3: studies, 7-11) (Tables 1 and 2).

There has been no persuasive cost-benefit analysis of accreditation internationally, and we intend to address this oversight in aim 2 via study 6. Insufficient work has been directed towards the assessment of new methods of accreditation, such as short-notice surveys (i.e., testing their validity) and tracking patients on their journeys through the system, whereby services are assessed based on the quality of care delivered longitudinally. These initiatives require novel assessment methods, e.g., studies 9 and 10 (Table 2).

To address aim 4, we will develop and test a new standard for consumer involvement in accreditation, which will be required for the next generation of accreditation designs [23]. The research technologies, which we will use in unique configurations across the studies, include the Delphi method in study 1, following our use of an earlier version of this in previous research; [40] ethnographic mapping in study 3, based on our experience in recent research; [41] and randomized designs applied to health-service organizational research in studies 2, 5, and 12. A mix of studies of this kind is challenging to do, but is needed given the pervasiveness of accreditation and its lack of an evidence base. ACCREDIT results from the 12 studies will facilitate a systems view of accreditation; given its complexity, this seems highly desirable.

## Conclusion

The ACCREDIT project has been planned in response to questions that the partners, customers of accreditation services, policy bodies (e.g., ACSQHC and CEC), and public and private funders of health-care have raised for many years about the utility, reliability, and cost-effectiveness of accreditation. Our findings are designed to build on what we already know, fill a number of research gaps, and facilitate the improvement of accreditation and the transparency and credibility of the accreditation, surveying and standards-setting processes.

## Abbreviations

ACCREDIT: **A**ccreditation **C**ollaborative for the **C**onduct of **R**esearch, **E**valuation and **D**esignated **I**nvestigations through **T**eamwork; ACHS: The Australian Council on Healthcare Standards; ACSAA: Aged Care and Standards Accreditation Agency; ACSQHC: Australian Commission on Safety and Quality in Health Care; AGPAL: Australian General Practice Accreditation Limited; AIHI: Australian Institute of Health Innovation; CEC: Clinical Excellence Commission; ISO: International Organization for Standardization; MARQuIS: Methods of Assessing Response to Quality Improvement Strategies

## Competing interests

The authors declare that they have no competing interests.

## Authors' contributions

JB and JW are the Chief Investigators of the ACCREDIT project. JB, JW, BJ, SC, MBr, MBa, CH, DG and MP contributed to developing the overarching research strategy. AC, AG and JC are responsible for specified studies. MW provided statistical advice and expertise. DD supported the development of the research proposal and provided advice on securing ethics approval. DG is senior research fellow working across the projects and MP provides expert advice. RH and MM are respectively responsible for qualitative and quantitative research across the projects. JØ, CP, RS and CS are members of the International Advisory Group, and offered strategic counsel on the program of studies. Team members are responsible, respectively, for leading studies 5, 6 and 7 [JB]; study 12 [JW]; study 1 [BJ]; study 3 [SC]; study 2 [MBr]; study 7 [MBa]; study 9 [CH]; studies 4 and 11 [DG and MP]; study 8 [AC]; and study 10 [AG and JC]. All authors read and approved the final manuscript.
